# Radiosynthesis and Preclinical Evaluation of a Carbon-11 Labeled Phosphodiesterase 7 Inhibitor for PET Neuroimaging

**DOI:** 10.1021/acsmedchemlett.5c00385

**Published:** 2025-08-18

**Authors:** Zhiwei Xiao, Jiyun Sun, Masayuki Fujinaga, Huiyi Wei, Chunyu Zhao, Achi Haider, Richard Van, Shi Kuang, Tomoteru Yamasaki, Yiding Zhang, Jian Rong, Kuan Hu, Jiahui Chen, Erick Calderon Leon, Wakana Mori, Lin Xie, Junjie Wei, Yi Xu, Yihan Shao, Han-Ting Zhang, Ying Xu, Chongzhao Ran, KC Kent Lloyd, Lu Wang, Ming-Rong Zhang, Steven H. Liang

**Affiliations:** 1 Department of Radiology, Division of Nuclear Medicine and Molecular Imaging, Massachusetts General Hospital and Harvard Medical School, 55 Fruit Street, Boston, Massachusetts 02114, United States; 2 Department of Advanced Nuclear Medicine Sciences, National Institute of Radiological Sciences, 13520 National Institutes for Quantum and Radiological Science and Technology, Chiba 263-8555, Japan; 3 Center of Cyclotron and PET Radiopharmaceuticals, Department of Nuclear Medicine, and PET/CT-MRI Center, The First Affiliated Hospital of Jinan University, Guangzhou 510630, China; 4 Department of Chemistry and Biochemistry, 6187 University of Oklahoma, Norman, Oklahoma 73019, United States; 5 Departments of Neuroscience, Behavioral Medicine & Psychiatry, Physiology, and Pharmacology, the Rockefeller Neuroscience Institute, West Virginia University Health Sciences Center, Morgantown, West Virginia 26506, United States; 6 Department of Anesthesiology, 242612 Rutgers, The State University of New Jersey, Newark, New Jersey 07103, United States; 7 Department of Surgery, School of Medicine, and Mouse Biology Program, University of California, Davis, 2795 Second Street, Suite 400, Davis, California 95618, United States; 8 Athinoula A. Martinos Center for Biomedical Imaging, Department of Radiology, Massachusetts General Hospital and Harvard Medical School, Boston, Massachusetts 02114, United States; 9 Department of Cardiology, The First Affiliated Hospital of Jinan University, Guangzhou 510630, China

**Keywords:** Phosphodiesterase 7, Inhibitor, Quinazolinone scaffold, Positron emission tomography, Radioligand

## Abstract

Dysfunction of cyclic nucleotide phosphodiesterase 7 (PDE7) has been associated with excess intracellular cAMP concentrations, fueling pathogenic processes that are implicated in neurodegenerative disorders. This study aimed to develop a suitable positron emission tomography (PET) probe that allows noninvasive mapping of PDE7 in the mammalian brain. Based on a spiro cyclohexane-1,4′-quinazolinone scaffold with known inhibitory properties toward PDE7, we designed and synthesized a carbon-11 labeling tolerant methoxy analog. The resulting PET probe, code named [^11^C]­P7-2104 (**27**), was synthesized in high molar activities (170–220 GBq/μmol) with decay-corrected radiochemical yields of 34 ± 7%. *In vitro* cell uptake of [^11^C]**27** was 6–7-fold higher in PDE7 overexpressing cells compared to the controls, whereas an *in vitro* specificity of up to 90% was measured. *Ex vivo* metabolite studies revealed a high fraction of intact parent in the rat brain (98% at 5 min and 75% at 30 min postinjection). Considerable brain penetration was further corroborated by *ex vivo* biodistribution and PET imaging studies, the latter showing heterogenic brain uptake. While marginal blockade was observed by PET studies in rodents, a moderate, but dose-dependent, blockade was observed in the non-human primate brain following pretreatment with nonradioactive **27**. Accordingly, [^11^C]**27** will serve as a valuable lead compound for the development of a new arsenal of PDE7-targeted probes.

## Graphical abstract

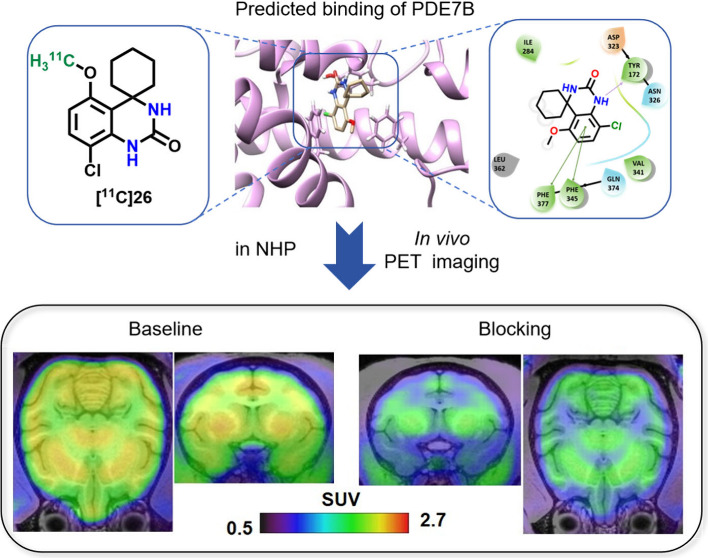

## Introduction

The intracellular second messengers, cyclic adenosine monophosphate (cAMP) and cyclic guanosine monophosphate (cGMP), play key roles in signal transduction involving a myriad of processes in the central nervous system (CNS) and immune system, as well as the cardiovascular system.^1–4^ Cyclic nucleotide phosphodiesterases (PDEs) constitute a superfamily of enzymes that degrade cAMP and cGMP. Human PDEs are derived from 21 genes and classified into 11 families (PDE1–11) according to the sequence homology of the C-terminal catalytic domain. Based on the substrate preferences, PDE families are grouped into cAMP-specific PDEs (4, 7, and 8), cGMP-specific PDEs (5, 6, and 9), and dual cAMP/cGMP-specific PDEs (1–3, 10, and 11).^5,6^ Among the cAMP-specific PDEs, PDE7 has recently gained increasing attention due to the broad implications in pro-inflammatory processes that involve T cell activation.^7^ Of note, the PDE7 family can be subdivided into PDE7A and PDE7B subunits.^8,9^ In terms of organ distribution, PDE7 was found to be primarily expressed in the brain and heart, followed by the liver, skeletal muscles, kidneys, testes, and pancreas. In particular, PDE7B is highly expressed in the brain in which the highest mRNA level was found in the striatum and the lowest in the cerebellum.^9^ On a cellular level, PDE7 isozymes are abundant in a variety of inflammatory and immune cells.^9–12^ It is worth mentioning that increased PDE7B expression was found in chronic lymphocytic leukemia cells.^13^

Intracellular cAMP levels are highly regulated and typically maintained in a narrow range for normal physiological functions. Indeed, PDE7 is considered to be crucial in maintaining homeostatic cAMP levels. Similarly, inhibition of PDE7 emerged as a promising therapeutic strategy for a variety of neurological, inflammatory, and immunological disorders where intracellular cAMP concentration is perturbed.^14,15^ A growing body of evidence suggested that inhibition of PDE7 might provide neuroprotective effects and be beneficial to the treatment of neurological disorders, especially neurodegenerative diseases, such as Alzheimer’s disease (AD),^16,17^ Parkinson’s disease (PD),^18^ and multiple sclerosis (MS).^19^ Because the second messenger cAMP not only mediates the signal transduction of the intracellular inflammatory mediators but also involves lymphocyte proliferation and the immune response with respect to cytokine/chemokine production,^20,21^ PDE7 inhibitors harbor the potential for the treatment of inflammatory conditions, such as autoimmune diseases (AIDs), autoimmune hepatitis (AIH), and rheumatoid arthritis (RA).^15,22–26^

In view of the potential of PDE7 as a drug target in neurological, inflammatory, and immunological disorders, the development of PDE7 inhibitors has progressed well in recent years (Figure 1). In terms of chemical structure, heterocyclic compounds, which have been summarized in previous reviews,^27,28^ have received considerable attention. Indeed, quinazolinone, quinazolinethione,^29^ and purine-2,6-dione derivatives^20,30^ with PDE7 inhibitory properties have been developed and evaluated. In addition, dual-selective PDE4/PDE7,^20,31–33^ PDE7/PDE8, and PDE7/GSK3 inhibitors^28^ were synthesized and assessed in animal models of neurological and peripheral inflammatory diseases. To the best of our knowledge, no PDE7-selective inhibitors are currently on the market or in clinical trials.^1^ Given that PDE7 shares a high degree of sequence homology with PDE4, most PDE7 inhibitors exert pharmacological activity against PDE4. Thus, the development of selective PDE7 inhibitors remains challenging.

**Figure 1:**
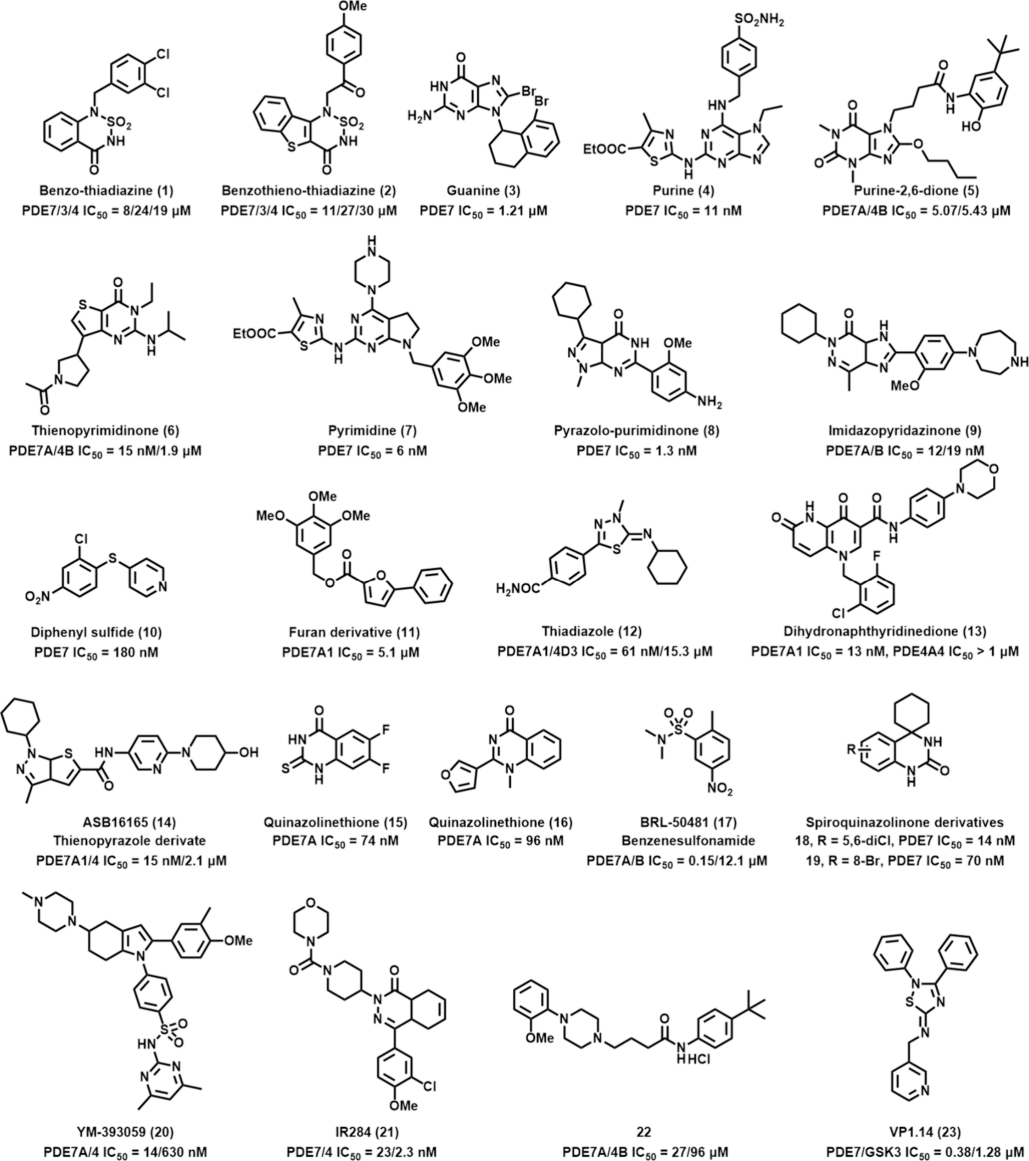
Selected compounds with PDE7 inhibitory properties.

Positron Emission Tomography (PET) is a noninvasive imaging modality widely used in preclinical and clinical settings due to its high sensitivity.^34,35^ As such, PET is ideally suited for early disease diagnosis, thereby providing prognostic information and allowing therapy monitoring. Further, PET can provide pharmacokinetic and pharmacodynamic information, which is crucial in early drug development to avoid late-stage failure of drug candidates.^36,37^ The potential of PDE7 as a therapeutic target has been elucidated for several years;^38^ however, only a few PDE7 radioligands for PET neuroimaging have been reported to date. Recently, a PDE7-targeted PET radioligand, code named [^11^C]­MTP38 (Figure 2), was advanced to humans.^39^ In the preclinical PET imaging study in rats and monkeys, [^11^C]­MTP38 penetrated the BBB and rapidly accumulated in the striatum, while a lower tracer uptake was found in the cerebellum.^40^ Although only moderate *in vivo* binding specificity was observed, potentially attributed to relatively fast clearance, [^11^C]­MTP38 served as a lead PET ligand scaffold to image PDE7 in the brain. Thomae et al. performed the synthesis and evaluation of [^18^F]­MICA-003 in mice (Figure 2).^41^ Although [^18^F]­MICA-003 exhibited a high affinity to PDE7 (17 nM) and readily crosses the blood–brain barrier, the rapid degradation to 2-[^18^F]­fluoroethanol^42^ hampered its further development. Our study hypothesized that the modification of the metabolically labile fluoroethyl group in the chemical structure of [^18^F]­MICA-003 would eliminate the generation of [^18^F]­fluoroethanol *in vivo* and provide a promising concept for the development of suitable PDE7-targeted PET ligands. Accordingly, based on the same spiro quinazoline structure, a ^18^F-isotopologue, [^18^F]­P7-2302, was developed with high affinity (0.18 nM) and selectivity to PDE7.^43^ Herein, we described the synthesis and preliminary pharmacology and ADME evaluation of ^11^C-isotopologue labeled compound **27** (code name P7-2104; Figure 2), followed by PET imaging in rodents and non-human primates (NHPs), the results of which might provide an excellent chemical phenotype in medicinal chemistry aimed for further PDE7 PET ligand development.

**Figure 2:**
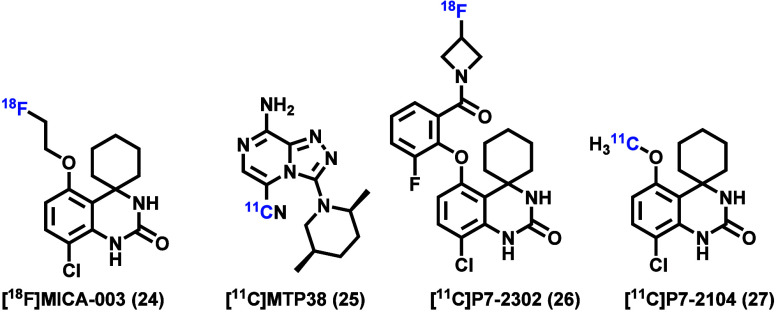
Reported PDE7 PET radioligands.

## Results and Discussion

Target compound **27** and the corresponding phenolic precursor **31** were efficiently synthesized in 2–3 steps from commercially available 2-chloro-5-methoxyaniline (**28**) with 49.5% and 12.4% overall yields (Scheme S1), respectively.

Compound **27**, discovered by Pfizer Research,^44^ exhibited equipotency toward PDE7 subtypes, namely, PDE7A and PDE7B. Therefore, we only measured PDE7A to confirm the binding potency. Specifically, by *in vitro* assessment, compound **27** exhibited an inhibitory constant (IC_50_) of 31 nM toward PDE7A (Figure 3). Further, the target compound was found to be selective over other PDE isomers (% inhibition <50% at 3 μM of compound **27**).

**Figure 3:**
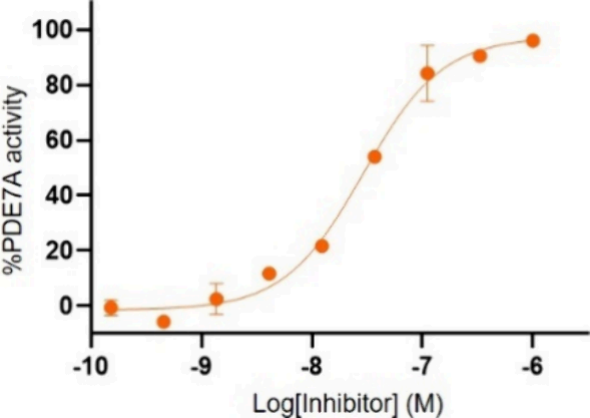
Concentration–response curve of compound **27** for inhibition of PDE7A activity.

Molecular docking was then performed with a homology model of PDE7B to explore potential interactions. The binding pocket is mainly hydrophobic, and major ligand–protein interactions were observed with Tyr172, Phe345, and Phe377 (Figure 4A). Indeed, target compound **27** formed a hydrogen bond of 2.306 Å with Tyr172. Further, π–π stacking was observed between the ligand and Phe345/377 (Figure 4B).

**Figure 4:**
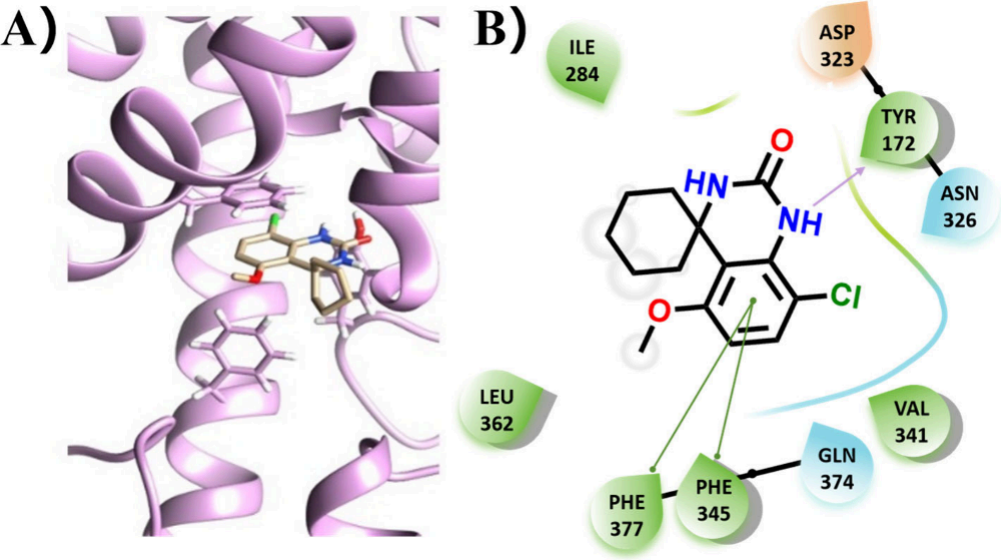
Docking position of compound **27** was generated using AutoDock Vina module of Chimera and Maestro. (A) AutoDock Vina (Chimera) generated model of compound **27** and (B) corresponding Maestro generated interaction plot, where purple arrow represents a hydrogen bond and green arrows highlight π–π stacking.

By *in vitro* safety studies, compound **27** did not exert an inhibitory effect on the hERG channel (IC_50_ > 100 μM). Except for weak inhibitory activity against CYP1A2 (IC_50_ = 4.4 μM), compound **27** did not markedly interact with any CYP450 isoforms (IC_50_ ≥ 10 μM).

NeuroPK study showed brain-to-plasma ratios (Kp) in the range of 3–5 at all measured time points, indicating that the concentration of compound **27** was higher in the brain than in the plasma throughout the study (Table 1 and Figure 5). While initially peaking at 3316.17 ± 378.14 ng/g (5 min), a steady washout was observed at later time points, suggesting fast and reversible kinetics. Indeed, less than 5% of the compound remained in the brain at the last measured time point (60 min). A similar trend was found in the plasma from 712.49 ± 153.23 ng/mL (5 min) to 39.39 ± 4.44 ng/mL (60 min).

**Table 1:** Rat PK Data in Plasma and Brain for Compound **27** ^a^

	plasma concentration (ng/mL)		brain concentration (ng/g)		Kp	
time (min)	mean	SD	mean	SD	mean	SD
5	712.49	153.23	3316.17	378.14	4.76	0.89
25	**229.06**	64.05	**876.42**	168.02	**4.00**	1.11
60	**39.39**	4.44	**131.68**	22.69	**3.35**	0.45

a The data was collected following a single intravenous administration to male Sprague–Dawley rats (1 mg/kg; *n* = 3); LLOQ: 5.10 ng/mL for plasma and 2.04 ng/mL for brain.

**Figure 5:**
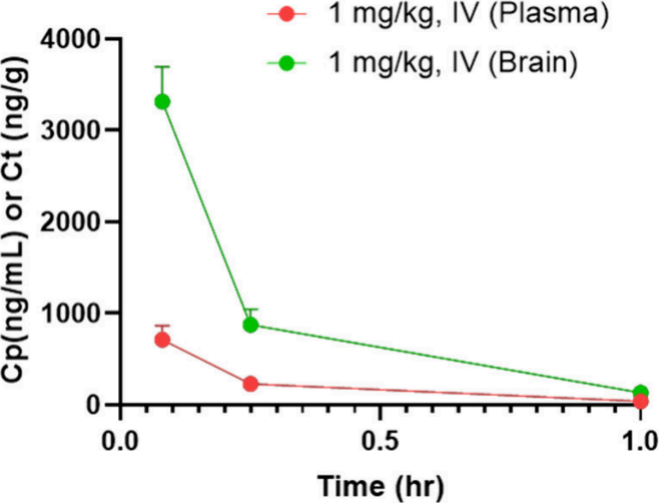
Plasma and brain concentration–time profiles (mean ± SD) of compound **27**.

[^11^C]**27** was synthesized in excellent radiochemical purity (>99%) and high molar activities (170–220 GBq/μmol) with decay-corrected radiochemical yields of 34 ± 7% (*n* = 12) relative to [^11^C]­CH_3_I (Figure 6A). [^11^C]**27** was reformulated in saline with less than 10% (v/v) ethanol as an injection solution. At three time points (30, 60, 90 min), the *in vitro* stability was tested, and no obvious degradation or radiolysis was found, which is shown in Figure 6B,C. The lipophilicity of [^11^C]**27** was evaluated by the “shake-flask” method, revealing a Log*D*_7.4_ value of 3.67 ± 0.04 (*n* = 3).

**Figure 6:**
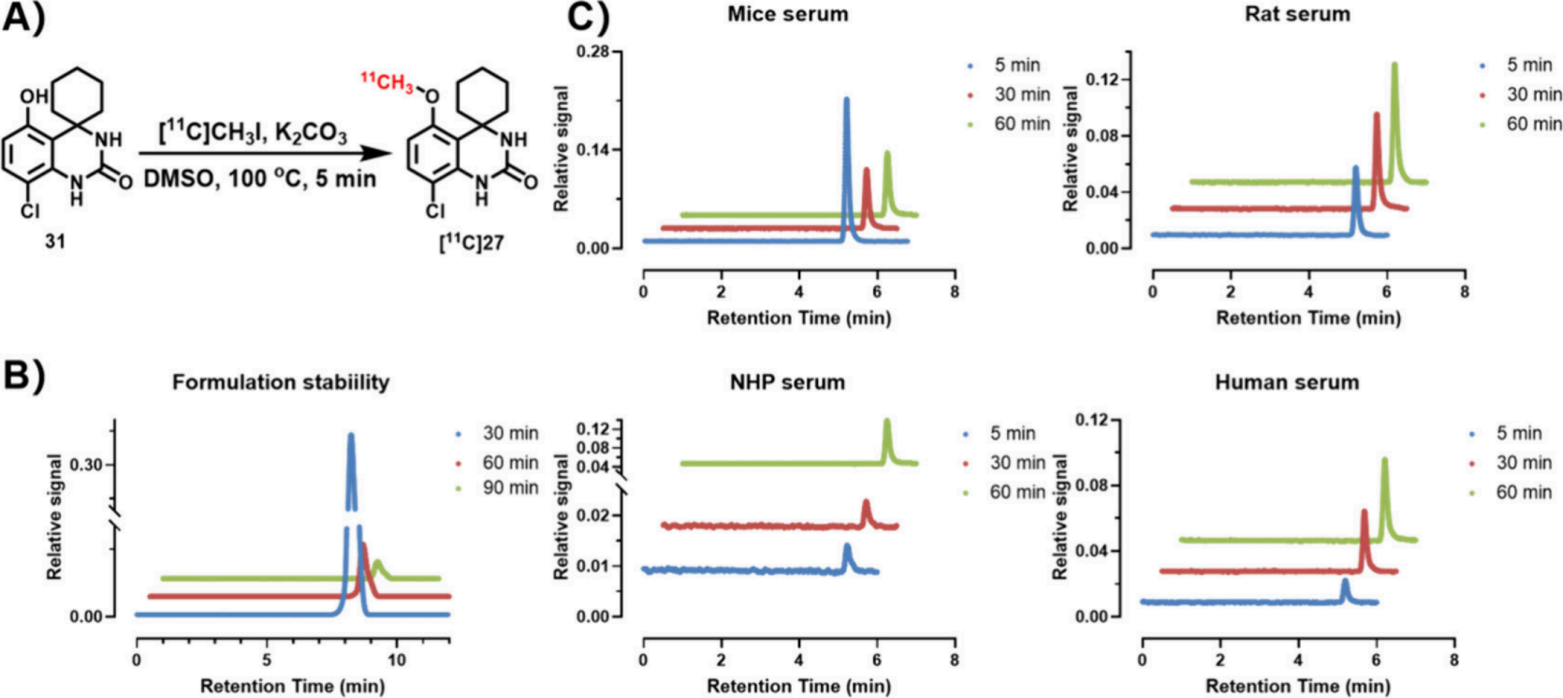
Carbon-11 labeling of [^11^C]**27** and in vitro stability studies: (A) Carbon-11 labeling conditions; (B) stability in PBS (containing 10% ethanol, v/v); (C) stability in serum of various species (rodents, NHPs, and humans).

Next, plasma protein binding was determined in mouse, rat, and human plasma. The results are presented as free fraction ratio in Figure 7A, which demonstrated a reasonable free fraction (≥1%) of **27** in the plasma. The *in vitro* cell uptake assay indicated that the radioactivity uptake of [^11^C]**27** was 6–7-fold higher in HEK293-PDE7B recombinant cell lines at 30 and 60 min incubation time (Figure 7B). The blocking study of [^11^C]**27** was carried out at 60 min of incubation time with unlabeled compound **27** (1 μM) and PDE7-targeted inhibitor BRL50481 (1 μM), demonstrating the specific binding (ca. 90% and 55% blockade, respectively) in cell uptake experiments (Figure 7C).

**Figure 7:**
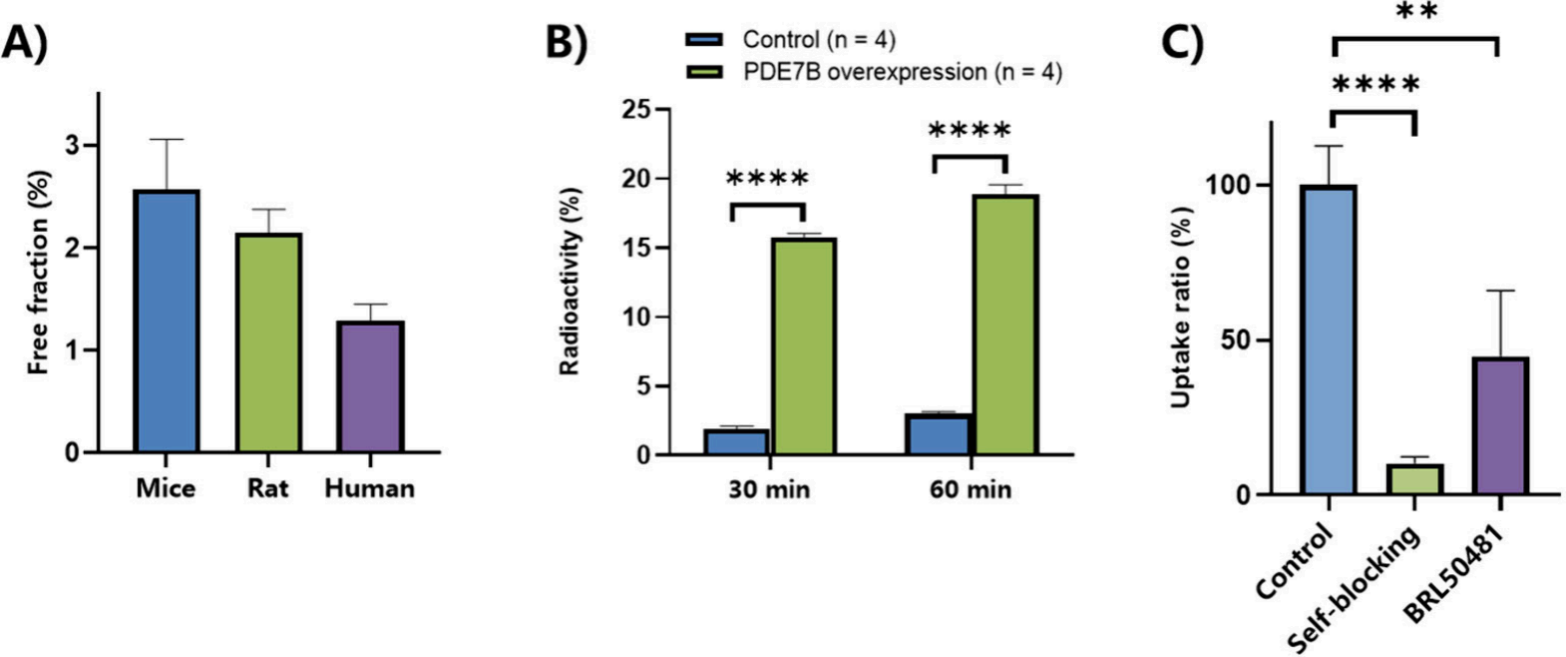
(A) Protein binding potency in mouse, rat, and human plasma; (B) Radioactivity uptake comparison between control and PDE7B overexpressing cells; (C) Blocking experiments in PDE7B overexpressing HEK293 cells using compound **27** and BRL50481 (*n* = 4).

To further evaluate the *in vivo* properties, [^11^C]**27** was injected into mice for microPET studies (1.85 MBq per mouse and 52–53 MBq per rat; molar activity 170–220 GBq/μmol). Time–activity curves (TACs) demonstrated that the tracer rapidly accumulated in the brain, whereas the peak SUV was reached within 2 min p.i. (see Supporting Information, Figure S1). In the meantime, a prolonged uptake was observed in liver (Figure S1D). Similarly, baseline and self-blocking PET experiments at a dose of 1 mg/kg were conducted in SD rats to confirm the *in vivo* specificity of [^11^C]**27**. In rats, the highest SUV values (>2) were reached at approximately 5 min postinjection, followed by a washout phase, ultimately reaching a plateau of 0.5 (SUV) at 20 min postinjection (Figure 8). [^11^C]**27** showed heterogeneous brain uptake, with the highest tracer uptake observed in the striatum and cortex, followed by the cerebellum, in the initial 10 min postinjection. Notably, however, tracer washout from the cerebellum was substantially slower than that in other brain regions. Pretreatment with nonradioactive compound **27** only showed marginally reduced brain uptake (see Supporting Information, Figure S2).

**Figure 8:**
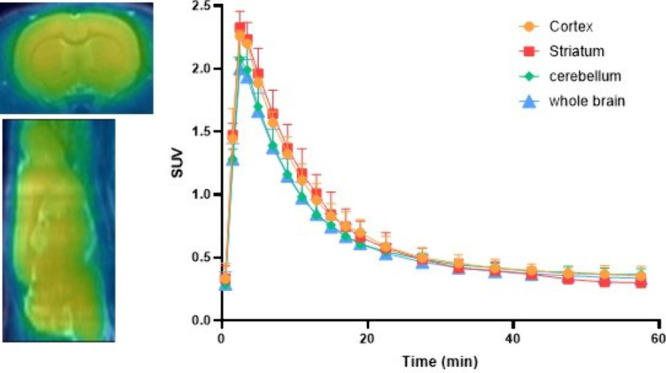
Summed PET images (0–20 min) in the rat brain following injection of [^11^C]**27** and the corresponding TACs of the cortex, striatum, cerebellum, and whole brain from 0 to 60 min.

Subsequently, [^11^C]**27** (200–278 MBq) was injected into monkeys under baseline and self-blocking conditions, and the summed PET images are presented in Figure 9. TACs indicated that [^11^C]**27** rapidly crossed the BBB and reached peak SUV (SUV_max_ = 3) at about 3 min p.i. Consistent with rat data, high tracer uptake was observed in PDE7B-rich monkey brain regions, including the cortex, putamen, and caudate, followed by relatively lower uptake in the cerebellum (Figure 9A). The radioactivity uptake in all assessed regions was moderately reduced by pretreatment with nonradioactive **27** at doses of 0.4 or 1 mg/kg (Figure 9B).

**Figure 9:**
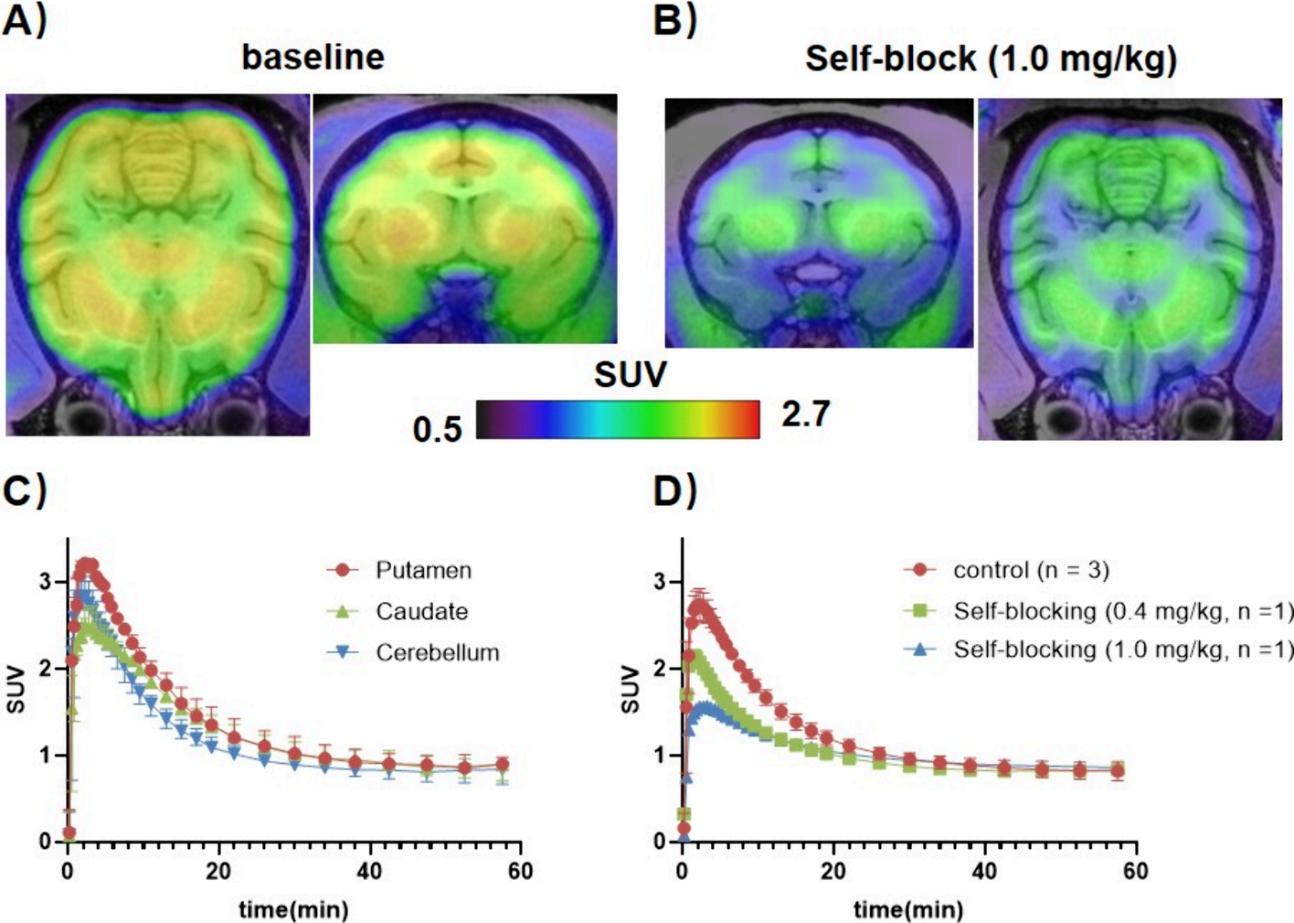
PET images and TACs in the monkey brain following the injection of [^11^C]**27**. (A) Representative whole-body PET images of the baseline scans, averaged from 0 to 20 min; (B) Whole-body PET images under self-blocking conditions (1.0 mg/kg), averaged at 0–20 min; (C) Whole brain TACs of the baseline scan from 0 to 60 min; (D) Whole brain TACs at different blocking doses.

To study the distribution of [^11^C]**27** in the whole body, *ex vivo* biodistribution analysis was carried out in CD-1 mice at four different time points (5, 15, 30, and 60 min postinjection, Figure 10). At 5 min p.i., the radioactivity was highly accumulated in the kidney, liver, brain, muscle, and particularly heart (up to 24% ID/g). Although the initial cardiac uptake was the highest, it decreased to less than 2% within 60 min. The radioactivity was rapidly washed out from the muscle, lung, kidney, and small intestine. In contrast, high retention in the liver was found at 60 min postinjection, suggesting a hepatobiliary clearance pathway.

**Figure 10:**
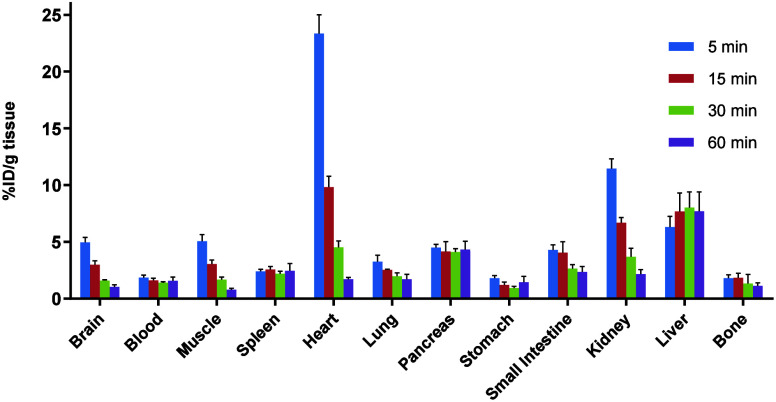
*Ex vivo* whole body biodistribution in CD-1 mice at four different time points (5, 15, 30, and 60 min) postinjection of [^11^C]**27**. The results are expressed as the percentage of the injected dose per gram of wet tissue (% ID/g).

Radiometabolite studies of [^11^C]**27** in rat brain and plasma (*n* = 2) were performed to evaluate *in vivo* stability (Figure 11). More than 98% intact [^11^C]**27** was found in the rat brains at 5 min. At 30 min postinjection, assessment of brain homogenates still showed more than 75% of the parent fraction. Compared to that of the brain, the clearance of [^11^C]**27** in plasma was substantially faster with 70% ± 18% and 19% ± 0.6% parent fraction found in the plasma at 5 and 30 min p.i., respectively.

**Figure 11:**
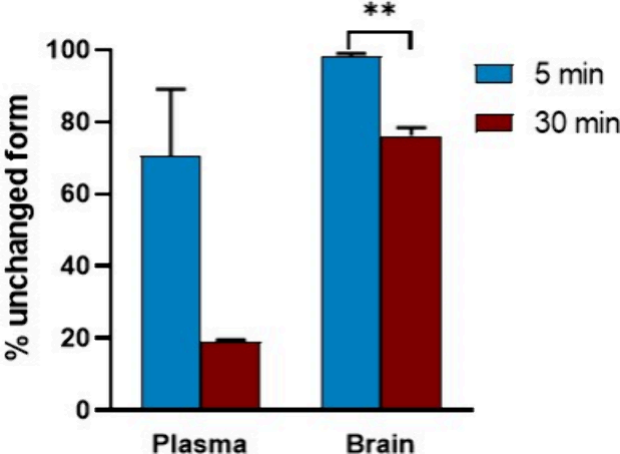
Radiometabolite study of [^11^C]**27** in rat brain and plasma (*n* = 2) at the time points of 5 and 30 min postinjection.

In this study, we report on the preclinical development of [^11^C]**27** as a potential PDE7-targeted PET radioligand. We were able to establish a simple and efficient synthetic route that allowed the synthesis of both reference compound and precursor in appropriate quality for radiochemistry and further biological assessment. Comparison between IC_50_ values of **27** toward PDE7 and other PDE isoenzymes demonstrated that compound **27** was highly potent and selective for PDE7. The former was supported by molecular docking analysis, revealing significant interactions between **27** and several amino acids in the hydrophobic binding pocket of PDE7. ADME parameters, including plasma protein binding, CYP450 profiling, and hERG channel assay indicated a beneficial safety profile, and NeuroPK studies in SD rats revealed that the brain-to-plasma ratio was >3 at all measured time points, indicating a high likelihood of brain penetration.

[^11^C]**27** was efficiently synthesized from its corresponding phenolic precursor **31** in one step using [^11^C]­CH_3_I, providing high radiochemical yields and high molar activity. Further, PDE7B overexpressing HEK293 cells^8,9^ were used to corroborate that [^11^C]**27** binds to the PDE7B isoform *in vitro*. [^11^C]**27** showed substantially increased cell uptake in PDE7B overexpressing versus control cells, whereas the *in vitro* specificity was confirmed by blocking studies with both unlabeled compound **27** and the PDE7 selective inhibitor BRL45081.

*In vivo* dynamic PET scans of [^11^C]**27** across different species (mice, rats, and monkeys) confirmed the anticipated brain penetration of this ligand. After intravenous administration, the radioactivity quickly permeated through the blood–brain barrier and was distributed heterogeneously in the brain. In rodents, tracer uptake was slightly higher in the striatum and cortex than in the cerebellum, particularly in the initial 10 min postinjection (Figure 8). Interestingly, there was a slower tracer washout from the cerebellum as compared to all other brain regions, indicating that there might be a distinct mechanism of retention in this particular region in rats. While initial tracer uptake was significantly higher in the NHP cerebellum, compared with the rat cerebellum, a faster tracer washout was observed from the NHP cerebellum. These results suggest that species differences in tracer kinetics are particularly pronounced in the cerebellum and that caution is warranted with regard to the extrapolation of rodent findings to higher species in this particular region. In addition, a dose-dependent signal reduction upon blocking experiments with nonradioactive **27** in the non-human primate (NHP) brain suggested that the tracer may be used to assess target occupancy in higher species (Figure 9). While similar clearance rates in brain and plasma were observed in rodent NeuroPK studies, the observation of similar washout kinetics does not necessarily exclude the presence of specific binding in the brain. Indeed, brain-to-plasma ratios (Kp) remained consistently elevated (>3) throughout the imaging period, indicating significant brain retention that exceeds passive diffusion or perfusion effects. This pattern is consistent with a tracer exhibiting rapid and reversible binding kinetics, potentially characterized by fast association and dissociation rates. Our non-human primate blocking studies further corroborate this interpretation, demonstrating a marked reduction in initial brain uptake upon PDE7 blockade while maintaining similar washout slopes, indicative of early target-specific binding in the brain. Notably, fast reversible kinetics can be advantageous for PET quantification, allowing the use of reversible compartmental models and facilitating accurate target occupancy measurements. Collectively, these data support that the PET signal shows, to some extent, specific and reversible binding to PDE7. Future optimization strategies will aim at reducing the dissociation kinetics and increasing the overall binding affinity of the candidate PET ligands. The results of whole-body *ex vivo* biodistribution in CD-1 mice were consistent with the PET findings. In consideration of the rapid washout from the kidneys and the sustained retention in the liver over 60 min (Figure 10), radioactivity may be excreted mainly via the hepatobiliary pathway. Indeed, although radiometabolite studies of [^11^C]**27** demonstrated high *in vivo* stability in the brain, radiometabolites were detected at 30 min postinjection in the plasma, which may explain the high liver uptake at later time points. Overall, we conclude that [^11^C]**27** shows promising performance characteristics *in vitro* but only limited *in vivo* potency. Further structural modifications, leading to an improved inhibitory constant in the single-digit nanomolar range, may improve the degree of specific binding *in vivo*.

## Conclusion

In summary, this work describes the *in vitro* and *in vivo* evaluation of PDE7-targeted PET radioligand [^11^C]**27** (code name [^11^C]­P7-2104), which is endowed with a promising chemical scaffold of spiro-cyclohexane-1,4′-quinazolinone. While target compound **27** proved to be potent and selective *in vitro*, cross-species *in vivo* studies with the radiolabeled analog, [^11^C]**27**, showed only moderately specific binding, thus warranting further structural modifications to achieve high *in vivo* specificity. The availability of suitable PDE7 PET radioligands is of critical value to understand the role of PDE7 in various disease pathologies, as well as to guide preclinical and clinical drug discovery efforts.

## Supplementary Material



## Notes

Methods for chemistry, molecular docking, in vitro inhibitory assay, CYP450 isoform metabolism profiling, pharmacokinetics in plasma and brain, radiochemistry, lipophilicity, plasma protein binding, in vitro cell uptake, PET imaging (blocking study), whole-body *ex vivo* biodistribution, radiometabolite analysis, and statistical analysis. Summed PET images and corresponding TACs in mouse and rat brains and mouse heart and liver (Figures S1 and S2), CYP450 profiling, and NMR spectra (PDF)

## 10

Department of Radiology and Imaging Sciences, Emory University, 1364 Clifton Road, Atlanta, Georgia 30322, United States

## Abbreviations

PDE7: phosphodiesterase 7
PET: positron emission tomography
NHP: non-human primate
cAMP: cyclic adenosine monophosphate
cGMP: cyclic guanosine monophosphate
CNS: central nervous system
AD: Alzheimer’s disease
PD: Parkinson’s disease
MS: multiple sclerosis
AID: autoimmune disease
AIH: autoimmune hepatitis
RA: rheumatoid arthritis
GSK3: glycogen synthase kinase-3
IC_50_: half-maximal inhibitory concentration
SUV: standard uptake value
LLOQ: lower limit of quantification

## References

[1] Baillie G. S., Tejeda G. S., Kelly M. P. (2019). Therapeutic targeting of 3′,5′-cyclic
nucleotide phosphodiesterases: inhibition and beyond. Nat. Rev. Drug Discovery.

[2] Kelly M. P. (2018). Cyclic
nucleotide signaling changes associated with normal aging and age
related diseases of the brain. Cell. Signal..

[3] Kim G. E., Kass D. A. (2016). Cardiac Phosphodiesterases
and Their Modulation for
Treating Heart Disease. Handb. Exp. Pharmacol..

[4] Ahmad F., Murata T., Shimizu K., Degerman E., Maurice D., Manganiello V. (2015). Cyclic nucleotide
phosphodiesterases: important signaling
modulators and therapeutic targets. Oral Dis..

[5] Maurice D. H., Palmer D., Tilley D. G., Dunkerley H. A., Netherton S. J., Raymond D. R., Elbatarny H. S., Jimmo S. L. (2003). Cyclic nucleotide phosphodiesterase activity, expression,
and targeting in cells of the cardiovascular system. Mol. Pharmacol..

[6] Kelly M. P., Adamowicz W., Bove S., Hartman A. J., Mariga A., Pathak G., Reinhart V., Romegialli A., Kleiman R. J. (2014). Select 3′,5′-cyclic nucleotide phosphodiesterases
exhibit altered expression in the aged rodent brain. Cell. Signal..

[7] Sun J., Xiao Z., Haider A., Gebhard C., Xu H., Luo H.-B., Zhang H.-T., Josephson L., Wang L., Liang S. H. (2021). Advances in Cyclic Nucleotide Phosphodiesterase-Targeted
PET Imaging and Drug Discovery. J. Med. Chem..

[8] Bloom T. J., Beavo J. A. (1996). Identification and
tissue-specific expression of PDE7
phosphodiesterase splice variants. Proc. Natl.
Acad. Sci. U. S. A..

[9] Reyes-Irisarri E., Perez-Torres S., Mengod G. (2005). Neuronal expression
of cAMP-specific
phosphodiesterase 7B mRNA in the rat brain. Neuroscience.

[10] Miro X., Perez-Torres S., Palacios J. M., Puigdomenech P., Mengod G. (2001). Differential distribution
of cAMP-specific phosphodiesterase
7A mRNA in rat brain and peripheral organs. Synapse.

[11] Gardner C., Robas N., Cawkill D., Fidock M. (2000). Cloning and characterization
of the human and mouse PDE7B, a novel cAMP-specific cyclic nucleotide
phosphodiesterase. Biochem. Biophys. Res. Commun..

[12] Han P., Zhu X., Michaeli T. (1997). Alternative
splicing of the high affinity cAMP-specific
phosphodiesterase (PDE7A) mRNA in human skeletal muscle and heart. J. Biol. Chem..

[13] Zhang L., Murray F., Zahno A., Kanter J. R., Chou D., Suda R., Fenlon M., Rassenti L., Cottam H., Kipps T. J., Insel P. A. (2008). Cyclic nucleotide
phosphodiesterase
profiling reveals increased expression of phosphodiesterase 7B in
chronic lymphocytic leukemia. Proc. Natl. Acad.
Sci. U. S. A..

[14] Safavi M., Baeeri M., Abdollahi M. (2013). New methods for the discovery and
synthesis of PDE7 inhibitors as new drugs for neurological and inflammatory
disorders. Expert Opin Drug Discov.

[15] Gonzalez-Garcia C., Bravo B., Ballester A., Gomez-Perez R., Eguiluz C., Redondo M., Martinez A., Gil C., Ballester S. (2013). Comparative assessment of PDE 4 and 7 inhibitors as
therapeutic agents in experimental autoimmune encephalomyelitis. Br. J. Pharmacol..

[16] Bartolome F., de la Cueva M., Pascual C., Antequera D., Fernandez T., Gil C., Martinez A., Carro E. (2018). Amyloid beta-induced
impairments on mitochondrial dynamics, hippocampal neurogenesis, and
memory are restored by phosphodiesterase 7 inhibition. Alzheimers Res. Ther.

[17] Perez-Gonzalez R., Pascual C., Antequera D., Bolos M., Redondo M., Perez D. I., Perez-Grijalba V., Krzyzanowska A., Sarasa M., Gil C., Ferrer I., Martinez A., Carro E. (2013). Phosphodiesterase 7 inhibitor reduced cognitive impairment and pathological
hallmarks in a mouse model of Alzheimer’s disease. Neurobiol Aging.

[18] Morales-Garcia J. A., Alonso-Gil S., Santos A., Perez-Castillo A. (2020). Phosphodiesterase
7 Regulation in Cellular and Rodent Models of Parkinson’s Disease. Mol. Neurobiol.

[19] Mestre L., Redondo M., Carrillo-Salinas F. J., Morales-Garcia J. A., Alonso-Gil S., Perez-Castillo A., Gil C., Martinez A., Guaza C. (2015). PDE7 inhibitor TC3.6 ameliorates
symptomatology in a model of primary
progressive multiple sclerosis. Br. J. Pharmacol..

[20] Chlon-Rzepa G., Jankowska A., Slusarczyk M., Swierczek A., Pociecha K., Wyska E., Bucki A., Gawalska A., Kolaczkowski M., Pawlowski M. (2018). Novel butanehydrazide derivatives
of purine-2,6-dione as dual PDE4/7 inhibitors with potential anti-inflammatory
activity: Design, synthesis and biological evaluation. Eur. J. Med. Chem..

[21] Jones N. A., Leport M., Holand T., Vos T., Morgan M., Fink M., Pruniaux M. P., Berthelier C., O’Connor B. J., Bertrand C., Page C. P. (2007). Phosphodiesterase
(PDE) 7 in inflammatory cells from patients with asthma and COPD. Pulm Pharmacol Ther.

[22] Szczypka M. (2020). Role of Phosphodiesterase
7 (PDE7) in T Cell Activity. Effects of Selective PDE7 Inhibitors
and Dual PDE4/7 Inhibitors on T Cell Functions. Int. J. Mol. Sci..

[23] Swierczek A., Pociecha K., Slusarczyk M., Chlon-Rzepa G., Bas S., Mlynarski J., Wieckowski K., Zadrozna M., Nowak B., Wyska E. (2020). Comparative
Assessment of the New PDE7 Inhibitor - GRMS-55 and Lisofylline
in Animal Models of Immune-Related Disorders: A PK/PD Modeling Approach. Pharm. Res..

[24] Redondo M., Palomo V., Brea J., Perez D. I., Martin-Alvarez R., Perez C., Paul-Fernandez N., Conde S., Cadavid M. I., Loza M. I., Mengod G., Martinez A., Gil C., Campillo N. E. (2012). Identification in
silico and experimental validation
of novel phosphodiesterase 7 inhibitors with efficacy in experimental
autoimmune encephalomyelitis mice. ACS Chem.
Neurosci..

[25] Redondo M., Brea J., Perez D. I., Soteras I., Val C., Perez C., Morales-Garcia J. A., Alonso-Gil S., Paul-Fernandez N., Martin-Alvarez R., Cadavid M. I., Loza M. I., Perez-Castillo A., Mengod G., Campillo N. E., Martinez A., Gil C. (2012). Effect of
phosphodiesterase 7 (PDE7) inhibitors in experimental autoimmune
encephalomyelitis mice. Discovery of a new chemically diverse family
of compounds. J. Med. Chem..

[26] Paterniti I., Mazzon E., Gil C., Impellizzeri D., Palomo V., Redondo M., Perez D. I., Esposito E., Martinez A., Cuzzocrea S. (2011). PDE 7 inhibitors:
new potential drugs
for the therapy of spinal cord injury. PLoS
One.

[27] PDE7 inhibitors. Expert Opinion on Therapeutic Patents 2002, 12, 601–603. 10.1517/13543776.12.4.601

[28] Jankowska A., Swierczek A., Chlon-Rzepa G., Pawlowski M., Wyska E. (2017). PDE7-Selective and Dual Inhibitors: Advances in Chemical and Biological
Research. Curr. Med. Chem..

[29] Elfeky S. M., Sobahi T. R., Gineinah M. M., Ahmed N. S. (2020). Synthesis, biological
screening, and molecular docking of quinazolinone and quinazolinethione
as phosphodiesterase 7 inhibitors. Arch Pharm.
(Weinheim).

[30] Wojcik-Pszczola K., Chlon-Rzepa G., Jankowska A., Ellen E., Swierczek A., Pociecha K., Koczurkiewicz P., Piska K., Gawedzka A., Wyska E., Knapik-Czajka M., Pekala E., Gosens R. (2019). Novel phosphodiesterases
inhibitors from the group of purine-2,6-dione derivatives as potent
modulators of airway smooth muscle cell remodelling. Eur. J. Pharmacol..

[31] Rucilova V., Swierczek A., Vanda D., Funk P., Lemrova B., Gawalska A., Bucki A., Nowak B., Zadrozna M., Pociecha K., Soural M., Wyska E., Pawlowski M., Chlon-Rzepa G., Zajdel P. (2021). New imidazopyridines with phosphodiesterase
4 and 7 inhibitory activity and their efficacy in animal models of
inflammatory and autoimmune diseases. Eur. J.
Med. Chem..

[32] Jankowska A., Satala G., Kolaczkowski M., Bucki A., Gluch-Lutwin M., Swierczek A., Pociecha K., Partyka A., Jastrzebska-Wiesek M., Lubelska A., Latacz G., Gawalska A., Bojarski A. J., Wyska E., Chlon-Rzepa G. (2020). Novel anilide and benzylamide derivatives
of arylpiperazinylalkanoic acids as 5-HT1A/5-HT7 receptor antagonists
and phosphodiesterase 4/7 inhibitors with procognitive and antidepressant
activity. Eur. J. Med. Chem..

[33] Sharma H., Lather V., Grewal A. S., Pandita D. (2019). Synthesis,
Anti-inflammatory
Activity and Docking Studies of Some Newer 1,3-Thiazolidine-2,4-dione
Derivatives as Dual Inhibitors of PDE4 and PDE7. Curr. Comput. Aided Drug Des.

[34] Rong J., Haider A., Jeppesen T. E., Josephson L., Liang S. H. (2023). Radiochemistry for positron emission
tomography. Nat. Commun..

[35] Deng X., Rong J., Wang L., Vasdev N., Zhang L., Josephson L., Liang S. H. (2019). Chemistry for Positron Emission Tomography:
Recent Advances in ^11^C-, ^18^F-, ^13^N-, and ^15^O-Labeling Reactions. Angew. Chem., Int. Ed..

[36] Sollini M., Cozzi L., Ninatti G., Antunovic L., Cavinato L., Chiti A., Kirienko M. (2021). PET/CT radiomics
in
breast cancer: Mind the step. Methods.

[37] Gambhir S. S. (2002). Molecular
imaging of cancer with positron emission tomography. Nat. Rev. Cancer.

[38] Sun J., Xiao Z., Haider A., Gebhard C., Xu H., Luo H.-B., Zhang H.-T., Josephson L., Wang L., Liang S. H. (2021). Advances in Cyclic Nucleotide Phosphodiesterase-Targeted
PET Imaging and Drug Discovery. J. Med. Chem..

[39] Kubota M., Seki C., Kimura Y., Takahata K., Shimada H., Takado Y., Matsuoka K., Tagai K., Sano Y., Yamamoto Y., Okada M., Kikuchi T., Ichise M., Kawamura K., Zhang M. R., Higuchi M. (2021). A first-in-human study
of (11)­C-MTP38, a novel PET ligand for phosphodiesterase 7. Eur. J. Nucl. Med. Mol. Imaging.

[40] Obokata N., Seki C., Hirata T., Maeda J., Ishii H., Nagai Y., Matsumura T., Takakuwa M., Fukuda H., Minamimoto T., Kawamura K., Zhang M.-R., Nakajima T., Saijo T., Higuchi M. (2020). Synthesis and preclinical evaluation
of [^11^C]­MTP38 as a novel PET ligand for phosphodiesterase
7 in the brain. bioRxiv.

[41] Thomae D., Servaes S., Vazquez N., Wyffels L., Dedeurwaerdere S., Van der Veken P., Joossens J., Augustyns K., Stroobants S., Staelens S. (2015). Synthesis and preclinical evaluation
of an ^18^F labeled PDE7 inhibitor for PET neuroimaging. Nucl. Med. Biol..

[42] Pan J., Pourghiasian M., Hundal N., Lau J., Bénard F., Dedhar S., Lin K.-S. (2013). 2-[^18^F]­Fluoroethanol and
3-[^18^F]­fluoropropanol: facile preparation, biodistribution
in mice, and their application as nucleophiles in the synthesis of
[^18^F]­fluoroalkyl aryl ester and ether PET tracers. Nuclear Medicine and Biology.

[43] Rong J., Zhao C., Chaudhary A. F., Jones E., Van R., Song Z., Li Y., Chen J., Zhou X., Patel J. S., Gao Y., Sun Z., Feng S., Zhang Z., Collier T. L., Ran C., Haider A., Shao Y., Yuan H., Liang S. H. (2025). Development
of a
Novel ^18^F-Labeled Radioligand for Imaging Phosphodiesterase
7 with Positron Emission Tomography. Mol. Pharmaceutics.

[44] Bernardelli P., Lorthiois E., Vergne F., Oliveira C., Mafroud A. K., Proust E., Pham N., Ducrot P., Moreau F., Idrissi M., Tertre A., Bertin B., Coupe M., Chevalier E., Descours A., Berlioz-Seux F., Berna P., Li M. (2004). Spiroquinazolinones as novel, potent,
and selective PDE7 inhibitors. Part 2: Optimization of 5,8-disubstituted
derivatives. Bioorg. Med. Chem. Lett..

