# Supplementary material for: Radiosynthesis and Preclinical Evaluation of a Carbon-11 Labeled Phosphodiesterase 7 Inhibitor for PET Neuroimaging
Source: ACS Med Chem Lett. 2025 Aug 18;16(9):1835–43. doi: 10.1021/acsmedchemlett.5c00385 (PMC12434536; doi:10.1021/acsmedchemlett.5c00385)
Supplement: Supplementary file 1 [file ml5c00385_si_001.pdf]

Supporting Information

Radiosynthesis and preclinical evaluation of a carbon-11 labeled PDE7 inhibitor for PET neuroimaging

Zhiwei Xiao,[a†] Jiyun Sun,[a†] Masayuki Fujinaga,[b†] Huiyi Wei,[c] Chunyu Zhao,[a] Achi Haider,[a] Richard Van,[d] Shi Kuang,[e] Tomoteru Yamasaki,[b] Yiding Zhang,[b] Jian Rong,[a] Kuan Hu,[b] Jiahui Chen,[a] Erick Calderon Leon,[d] Wakana Mori,[b] Lin Xie,[b] Junjie Wei,[c] Yi Xu,[f] Yihan Shao,[d] Han-Ting Zhang,[g] Chongzhao Ran,[e] KC Kent Lloyd,[h] Lu Wang,[c] Ming-Rong Zhang[b,*] and Steven H. Liang[a,*]

[a]Department of Radiology, Division of Nuclear Medicine and Molecular Imaging Massachusetts General Hospital and Harvard Medical School, 55 Fruit Street, Boston, Massachusetts 02114, United States

[b]Department of Advanced Nuclear Medicine Sciences, National Institute of Radiological Sciences, National Institutes for Quantum and Radiological Science and Technology, Chiba 263-8555, Japan

[c]Center of Cyclotron and PET Radiopharmaceuticals, Department of Nuclear Medicine and PET/CT-MRI Center, the First Affiliated Hospital of Jinan University, Guangzhou 510630, China

[d]Department of Chemistry and Biochemistry, University of Oklahoma, Norman, Oklahoma 73019, United States

[e]Athinoula A. Martinos Center for Biomedical Imaging, Department of Radiology, Massachusetts General Hospital and Harvard Medical School, Boston, Massachusetts, 02114, USA

[f]Department of Cardiology, The First Affiliated Hospital of Jinan University, Guangzhou, 510630, China.

[g]Departments of Neuroscience, Behavioral Medicine & Psychiatry, and Physiology &, and Pharmacology, the Rockefeller Neuroscience Institute, West Virginia University Health Sciences Center, Morgantown, WV 26506, United States.

[h]Department of Surgery, School of Medicine, and Mouse Biology Program, University of California, Davis, 2795 Second Street, Suite 400, Davis, CA 95618, USA.

General Information

All chemicals were purchased from commercial vendors and used without further purification unless otherwise indicated. Thin-layer chromatography (TLC) was conducted with 0.25 mm silica gel plates ($^{60}F_{254}$) and visualized by exposure to UV light (254 nm) or by staining with potassium permanganate. Column chromatography purification was performed using silica gel (SiliCycle Inc., 230−400 mesh, 40−63 μm). ^{1}H, ^{13}C, and ^{19}F NMR were obtained at 300, 75, and 282 MHz, respectively, on a Bruker spectrometer in $CDCl_3$ or d_6-DMSO solutions at room temperature, and the chemical shifts were quoted in δ values (parts per million, ppm) downfield relative to the internal TMS. The multiplicities are abbreviated as follows: s = singlet, d = doublet, t = triplet, q = quartet, m = multiplet, br = broad signal, and dd = doublet of doublets. For LC-MS, the ionization method is ESI using Agilent 6430 Triple Quad LC/MS. The animal experiments were approved by the Institutional Animal Care and Use Committee of Massachusetts General Hospital, National Institute of Radiological Sciences (Japan) and First Affiliated Hospital of Jinan University. C57BL/6 and CD1 (ICR) mice (female; 8 weeks, 20–25 g) and Sprague-Dawley rats (male; 8-9 weeks; 264-310 g) were kept on a 12 h light/12 h dark cycle and were allowed food and water *ad libitum*. Cynomolgus monkeys (Male, body weight 5.05 – 6.20 kg) used for PET/CT scan was deprived of food for 12 h but allowed to drink water at any time.

Chemistry

Target compound **27** and the corresponding demethylated precursor **31** were synthesized as a previous publication,[1] however, with minor modification, as shown in **Scheme 1**. Initial condensation between 2-chloro-5-methoxyaniline (**28**) and potassium isocyanate was accomplished under acidic conditions. In the presence of phosphorus pentoxide and methanesulfonic acid, intermediate **29** was reacted with cyclohexanone **30** to afford target compound **27** in 50% yield (over two steps). Precursor **31** was obtained through

demethylation of compound **26** using hydrobromic acid at a high temperature.

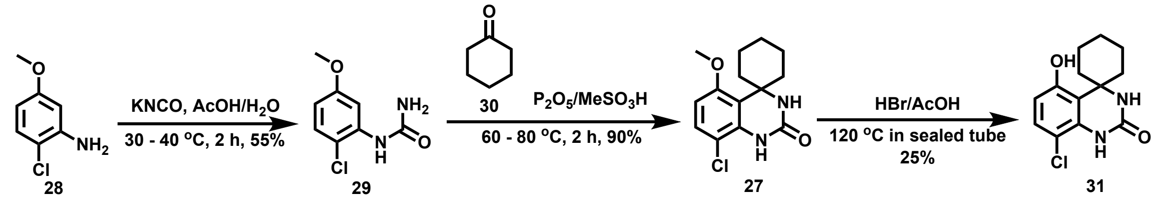

Scheme 1. Synthesis of compound **27** and the corresponding precursor **31** for ^{11}C labeling.

*8'-Chloro-5'-methoxy-1'H-spiro[cyclohexane-1,4'-quinazolin]-2'(3'H)-one (**27**)*

^{1}H NMR (300 MHz, CDCl$_3$) δ: 7.26 (s, 1H), 7.20 (d, *J* = 9.0 Hz, 1H), 7.09 (s, 1H), 6.51 (d, *J* = 9.0 Hz, 1H), 5.87 (s, 1H), 3.83 (s, 3H), 2.50 (td, *J* = 13.5, 4.4 Hz, 2H), 1.82 – 1.64 (m, 5H), 1.54 (qt, *J* = 14.0, 3.1 Hz, 2H), 1.30 (dddd, *J* = 16.5, 12.8, 8.3, 3.8 Hz, 1H). **^{13}C NMR (75 MHz, CDCl$_3$) δ:** 155.7, 151.3, 132.7, 128.5, 115.0, 111.3, 106.4, 58.2, 55.6, 35.2, 24.5, 20.7. **LCMS:** *m/z* = 281.1 (M+H$^+$).

*8'-Chloro-5'-hydroxy-1'H-spiro[cyclohexane-1,4'-quinazolin]-2'(3'H)-one (**31**)*

^{1}H NMR (300 MHz, CDCl$_3$) δ: 7.08 (d, *J* = 8.6 Hz, 2H), 6.37 (d, *J* = 8.7 Hz, 1H), 2.60 (td, *J* = 13.3, 4.1 Hz, 2H), 1.86 – 1.70 (m, 5H), 1.61 – 1.42 (m, 2H), 1.40 – 1.29 (m, 1H). **^{13}C NMR (75 MHz, CDCl$_3$) δ:** 151.8, 151.4, 128.5, 127.5 (d, *J* = 1.1 Hz), 113.7, 111.3, 110.7, 57.9, 35.1, 24.4, 20.7. **LCMS:** *m/z* = 267.1 (M+H$^+$).

Molecular Docking

The structure of compound **27** was generated using ChemDraw and IQmol. Since no crystal structure of PDE7B was available, a homology model was constructed using Swiss-Model.[2, 3] The sequence of PDE7B was obtained from universal protein resource (uniprot.org) and a template structure (PDB 3G3N) was identified. After obtaining both the protein and ligand structures, they were docked globally by having the search box encompass the entire PDE7B protein and using AutoDock Vina in UCSF Chimera. The results for the

protein-ligand complex were saved and were then loaded into Schrödinger Maestro to generate the ligand interaction plot.

In vitro inhibitory assay to PDE7 of compound 27 and selectivity to other PDEs

Functional activity of target compound **27** for inhibition of PDE7A activity and the selectivity over other PDE isozymes (PDE1A/B/C, 2A, 3A/B, 4A/B/C/D, 5A, 8A, 9A, and 10A) were evaluated by Eurofins Panlabs Discovery Services and Reaction Biology Corp., respectively. Human recombinant enzymes were used for the assays. The inhibitory assays were performed and calculated in duplicate at 3 μM. If the inhibition percentage was less than 50%, the assay for half-maximal inhibitory concentration (IC_{50}) value was conducted in duplicate.

CYP450 isoform metabolism and *in vitro* safety profiling

The inhibitory constant of compound **26** toward cytochrome P450-dependent metabolic pathways was determined for individual CYP isoform (CYP1A2, CYP2C9, CYP2C19, CYP2D6, and CYP3A4). Reference inhibitors were included as positive controls. A 10 μM solution of compound **27** was used for initial screening. If the inhibition percentage was less than 50%, the experiment was stopped. If the inhibition potency was greater than 50%, the gradient concentrations of compound **27** were used for IC_{50} determination. The P450 reactions were detected at Ext/Em: 405 nm/460 nm (530 nm/605 nm for CYP2C9) in a

time-dependent manner for 80 min with an acquisition interval of 2 min. IC_{50} curves were plotted using a non-linear regression model with Graphpad Prism 6. The latter method was further applied to assess the inhibitory constant of compound **27** to the hERG channel.

Pharmacokinetics in Plasma and Brain (NeuroPK study)

Nine male wildtype Sprague Dawley rats were administered a bolus injection containing compound **27** in saline (1 mg/kg, 2 mL/kg) via the tail vein. Blood samples (approximately 120 μL) were harvested under light isoflurane anesthesia from a set of three rats at 0.08, 0.25, and 1 hr. Blood and brain samples were collected at 5, 25 and 60 min. Following centrifugation of blood samples, the resulting plasma supernatant was stored at -70 ± 10 ºC till further analysis. Brain samples were homogenized using ice-cold phosphate buffer saline (pH 7.4) and homogenates were stored below -70 ± 10 ºC until further analysis. Total homogenate volume was three times the brain weight. Plasma and brain samples were quantified by LC-MS/MS method. The plasma and brain concentration-time data of compound **27** was used for pharmacokinetic analysis.

Radiochemistry

Carbon-11 labeling of compound **27** was achieved by radiomethylation of phenolic precursor **31** in the presence of potassium carbonate. The synthetic process involving radiolabeling, purification and formulation, was conducted using an automated module in an

overall synthesis time of 60 min from the end of bombardment. [^{11}C]CH_3I was synthesized from cyclotron-produced [^{11}C]CO_2, which was obtained via the $^{14}N(p,\alpha)^{11}C$ nuclear reaction. Briefly, [^{11}C]CO_2 was bubbled into a solution of $LiAlH_4$ (0.4 M in THF, 300 μL). After evaporation, the resulting reaction mixture was treated with hydroiodic acid (57% aqueous solution, 300 μL). [^{11}C]CH_3I was transferred under helium gas with heating into a reaction vessel containing a solution of the precursor (1.0 mg) in anhydrous dimethyl sulfoxide (DMSO, 300 μL) with 1.0 mg K_2CO_3. After the radioactivity reached a plateau during the transfer, the reaction vessel was warmed to 100 °C and maintained for 5 min. The mobile phase (2.5 mL) and H_2O (1.5 mL) were added to the reaction mixture, which was then injected into a semi-preparative HPLC system. HPLC purification was completed on a Phenomenex Luna 5μm C18 column,10 mm i.d. × 250 mm, UV at 254 nm, CH_3CN/H_2O = 50/50, 0.1%Et_3N, flow = 5 mL/min. The radioactive fraction corresponding to the desired product was collected in a sterile flask, diluted with 30 mL of water, and trapped on a Sep-Pak light C18 cartridge. After washing with 10 mL of water, the product was eluted from the C18 cartridge with 0.3 mL of ethanol and formulated with 6 mL of saline. The radiochemical purities and molar activity were measured by analytical HPLC (Xselect Hss T3, 4.6 mm i.d. × 150 mm, UV at 254 nm, CH_3CN/H_2O = 50/50, 0.1% Et_3N, flow = 1 mL/min). The identity of [^{11}C]**27** was confirmed by co-injection with the unlabeled standard.

Lipophilicity

The general procedure for lipophilicity measurement was previously described, [4] with minor modification in this work. Briefly, the measurement of Log*D* value was carried out by mixing [^{11}C]**27** (radiochemical purity > 99%) with *n*-octanol (3.0 g) and PBS (0.1 M, 3.0 g) in a test tube. Both *n*-octanol and PBS were pre-saturated with each other prior to use. The tube was first vortexed for 5 min, followed by centrifugation (~3500-4000 rpm) for an additional 5 min. PBS and *n*-octanol were aliquoted, weighed and the radioactivity in each component was measured using a Cobra Model 5002/5003 gamma counter. The Log*D* was determined by Log [ratio of radioactivity in *n*-octanol and aqueous layer, respectively] (n = 3).

Plasma protein binding

For the evaluation of plasma protein binding,[5] 55.5 MBq radiotracer was added to 150 µL of plasma, which was pre-incubated under 37 °C for 5 min (n = 3). The samples were incubated at 37 °C for 10 min. To each 150 µL of radiotracer-plasma solution was added 300 µL of ice-cold PBS and all samples were briefly vortexed. The samples were centrifuged at 14,000 g in Amicon centrifugal filters with a size cutoff of 10 kDa for 15 min at 4 °C, and the protein fraction was subsequently washed with 300 µL cold PBS at 21,000 g for 20 min at 4 °C. Additional cold PBS (300 µL) was used to wash the tube and collect all the filtrates. The radioactivity (in Becquerel) was measured in the protein fraction ($A_{protein}$), filtrate, and filter unit using a gamma counter (Wizard, PerkinElmer), A_{total} was calculated as the sum of radioactivity, and the free fraction f_u was calculated according to the following eq.: $f_u = 1 - A_{protein}/A_{total}$

In vitro cell uptake

Control study: HEK293-PDE7B Recombinant cells (catalog#60412, BPS Bioscience) and HEK293 control cells (catalog# CRL-1573, ATCC) – both in logarithmic growth phase – were plated in a 24 well plate (2 × 10^5 cells per well) and cultured overnight. After 24 hr, each well was added 2 μCi of [^{11}C]**27**, and incubated at 37 ^{o}C for 30 min or 60 min. The radioactive medium was aspirated and collected in a tube. The residual cells were washed twice with PBS, and then collected in the same tube. The cells were lysed with 1 N NaOH (200 μL) and washed twice with PBS, all the resulting solutions were collected in one tube. The radioactivity in the collected supernatant and cell lysis buffer (600 μL/tube) was measured using a Cobra Model 5002/5003 gamma counter, respectively.

Blocking study: HEK293-PDE7B cells in logarithmic growth phase were plated in 24 well plate (2 × 10^5 cells per well) and cultured overnight. After 24 h, each well was added 2 μCi of [^{11}C]**27** with or without unlabeled reference compound **27** or BRL50481, respectively. The resulting culture medium contained 2 μCi of [^{11}C]**27**, 1 μM of unlabeled compound **27** or BRL50481, and 5% DMSO. Cells were incubated at 37 ^{o}C for 60 min and samples were collected as described for the control study. The cell uptake was calculated according to the following eq.: $cell\ uptake\% = A_{lysis\ buffer}/(A_{lysis\ buffer}+A_{supernate})$ (n = 4)

PET imaging in rodents

All animal experiments were approved by the Institutional Animal Care and Use Committee of Massachusetts General Hospital or the National Institute of Radiological Sciences (Japan). C57BL/6 mice (female; 8 weeks, 20–25 g) and Sprague-Dawley rats (male; 8-9 weeks; 264-310 g) were kept on a 12 h light/12 h dark cycle and were allowed food and water *ad libitum*.

Rodent PET scans were carried out using a Genisys 4 PET (Sofie Biosciences, Culver, CA, USA) or a Siemens Inveon PET/CT system. Animals were kept under anesthesia using 1-2% (v/v) isoflurane in oxygen during the scan. The radiotracer (1.85 MBq for mice; 42-53 MBq for rats) was injected into the tail vein via a preinstalled catheter. A dynamic scan in 3D list mode was acquired for 60 min. The PET dynamic images were reconstructed using the manufacturer's acquisition software. Volumes of interest, including whole brain, cortex, striatum, hippocampus, thalamus and cerebellum were placed using AMIDE / PMOD software. The radioactivity was decay-corrected and expressed as standardized uptake value (SUV = radioactivity per mL tissue / injected radioactivity × bodyweight).

PET imaging in nonhuman primates

Cynomolgus monkeys used for PET/CT scan were deprived of food for 12h but allowed to drink water at any time. Animals (Male, bodyweight 5.0-5.2 kg) were anesthetized with ketamine, placed into the scanner (GE Discovery Elite 690, USA), and maintained with 2% isoflurane and 98% oxygen. A solution of [^{11}C]**27** (6.24–9.84 mCi) was injected into the monkey via a venous catheter, followed by a dynamic PET scan. For the blocking study, the blocking reagent reference compound **27** (0.4 or 1.0 mg/kg, iv) was used, followed by the injection of [^{11}C]**27**. Time-activity curves of each brain region were extracted from the corresponding VOIs and brain uptake was decay-corrected and expressed as SUV.

Whole-body *ex vivo* biodistribution studies in mice

A solution of [^{11}C]**27** (1.85 MBq/100 μL) was injected into CD-1 mice via the tail vein. Animals were sacrificed at 5, 15, 30, and 60 min post radiotracer injection (n= 4 for each time point). Major organs, including the whole brain, heart, liver, lungs, spleen, kidneys, small intestine (including contents), muscle, pancreas, stomach, bone, and blood were quickly harvested and weighed. The radioactivity in these tissues was measured using a Cobra Model 5002/5003 gamma counter, and all radioactivity measurements were decay corrected based on the half-life of carbon-11. The results are expressed as the percentage of injected dose per gram of wet tissue (%ID/g).

Radiometabolite Analysis

After intravenous injection of [^{11}C]**27** through the tail vein, SD rats were sacrificed at 5 min and 30 min post injection. Blood samples were collected and centrifuged at 14,000 rpm for 3 min at 4 °C to separate the plasma. The supernatant was collected and added to an ice-cooled test tube containing 100 μL of CH_3CN. After vortex for 10 s, the mixture was centrifuged at 14000 rpm for 3 min at 4 °C for deproteinization. The supernatant was collected, and the process was repeated until no precipitations were observed when CH_3CN was added. The rat brain was immediately dissected, homogenized with 400 μL of ice-cooled CH_3CN, and then centrifuged at 14,000 rpm for 5 min at 4 °C. The supernatant was collected in a test tube containing 100 μL of ice-cooled CH_3CN, and the process (vortex and centrifuge) was repeated until no precipitations were observed when CH_3CN was added. An aliquot of the supernatant (100 μL), obtained from the brain homogenate, was injected into and analyzed by a radio-HPLC system. The percentage of [^{11}C]**27** to total radioactivity (corrected for decay) on the HPLC charts was calculated as (peak area for [^{11}C]**27**/total peak area) × 100. The same procedure was used for metabolite analysis in plasma.

Statistical analysis

Statistical analysis was performed by Student's two-tailed *t*-test, and asterisks were used to indicate statistical significance: $^{*}p < 0.05$, $^{**}p \leq 0.01$, $^{***}p \leq 0.001$ and $^{****}p < 0.0001$.

Supporting figures

PET imaging

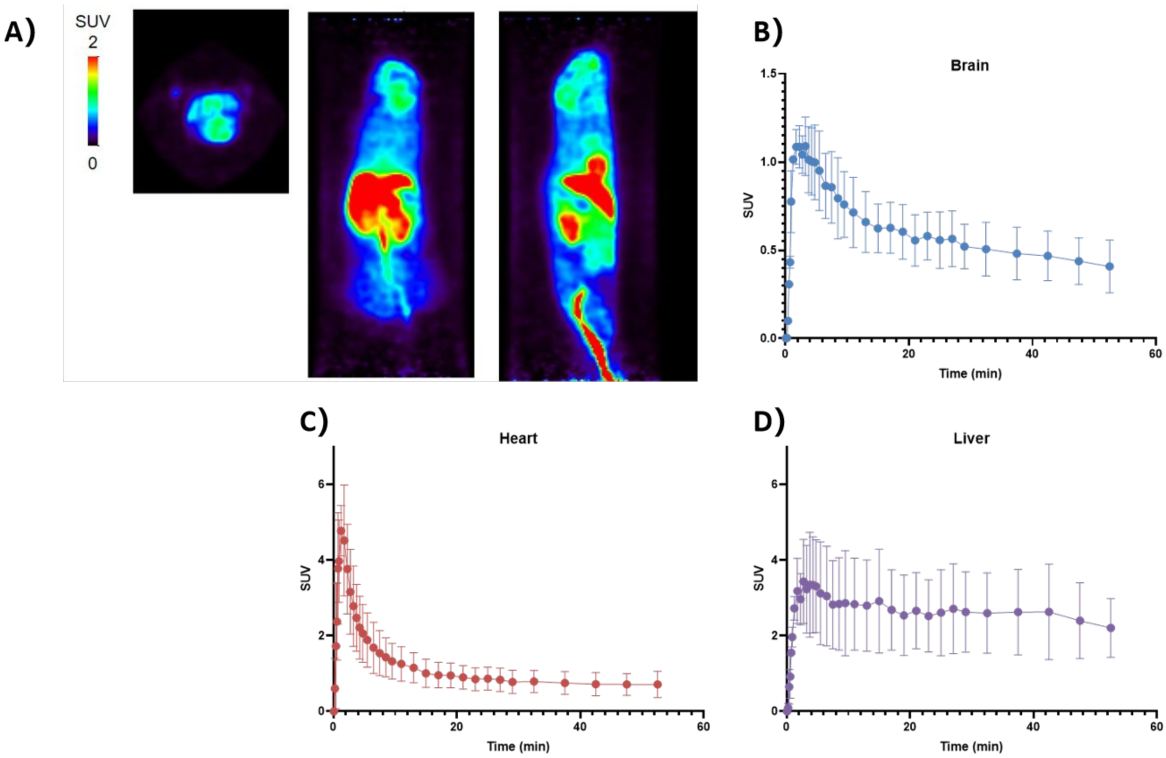

Figure S1. Summed PET images (0-20 min) in mice brain following injection of [^{11}C]**27** (A) and the corresponding TACs of whole brain, heart, and liver from 0 to 60 min (n = 2) (B, C, D).

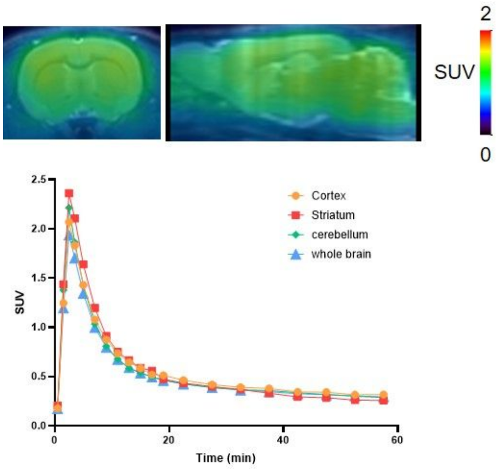

Figure S2. Summed PET images (0-20 min) in rat brain following co-injection of [^{11}C]**27** and compound **27** (1 mg/kg), the corresponding TACs of whole brain from 0 to 60 min.

CYP450 isoform metabolism profiling

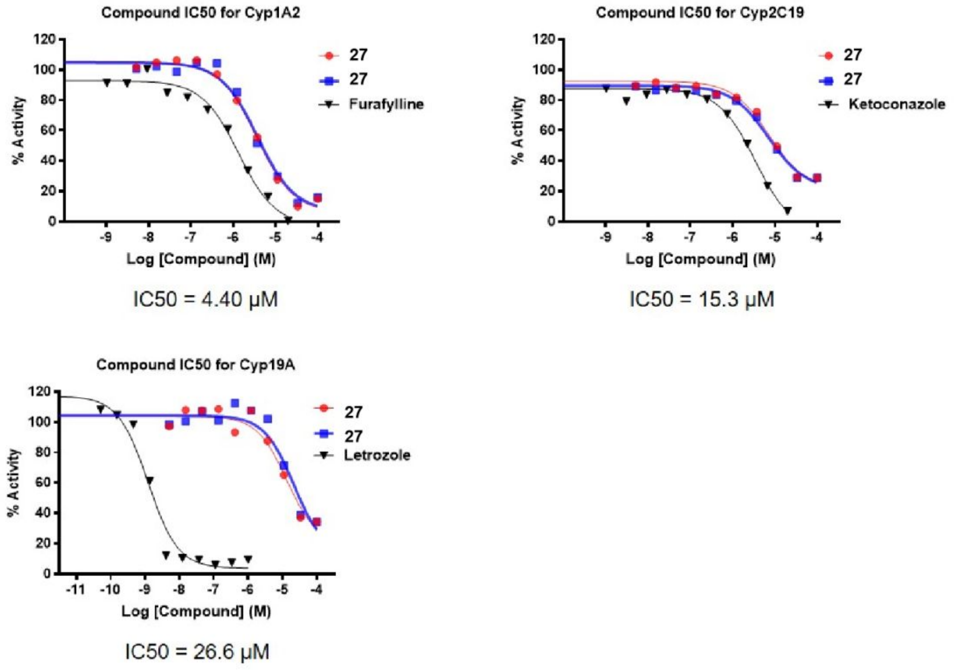

Figure S3. CYP450 profiling of compound **27** for individual CYP isoform (CYP1A2, CYP2C19 and CYP19A).

^{1}H spectrum of compound **27**

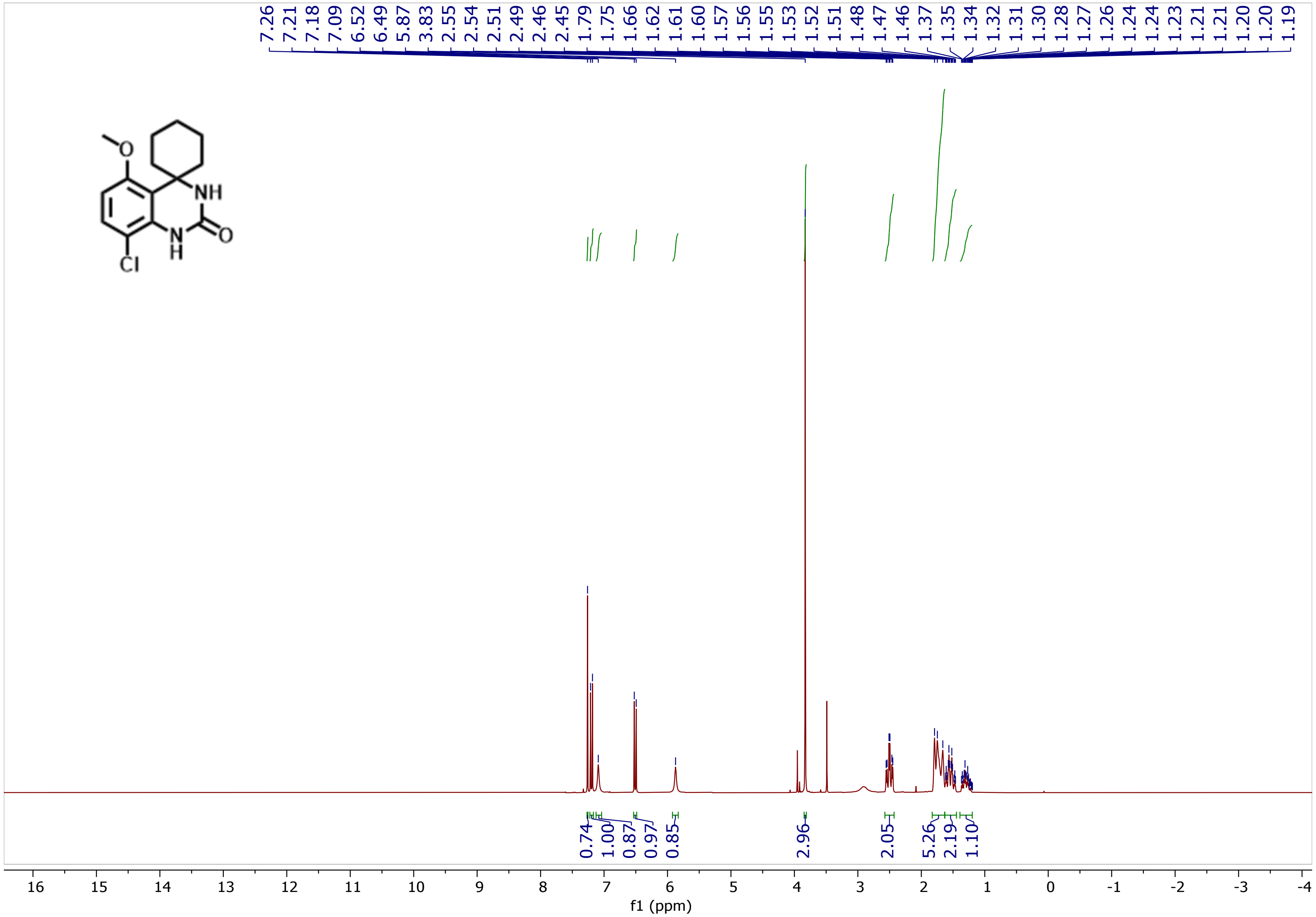

^{13}C spectrum of compound **27**

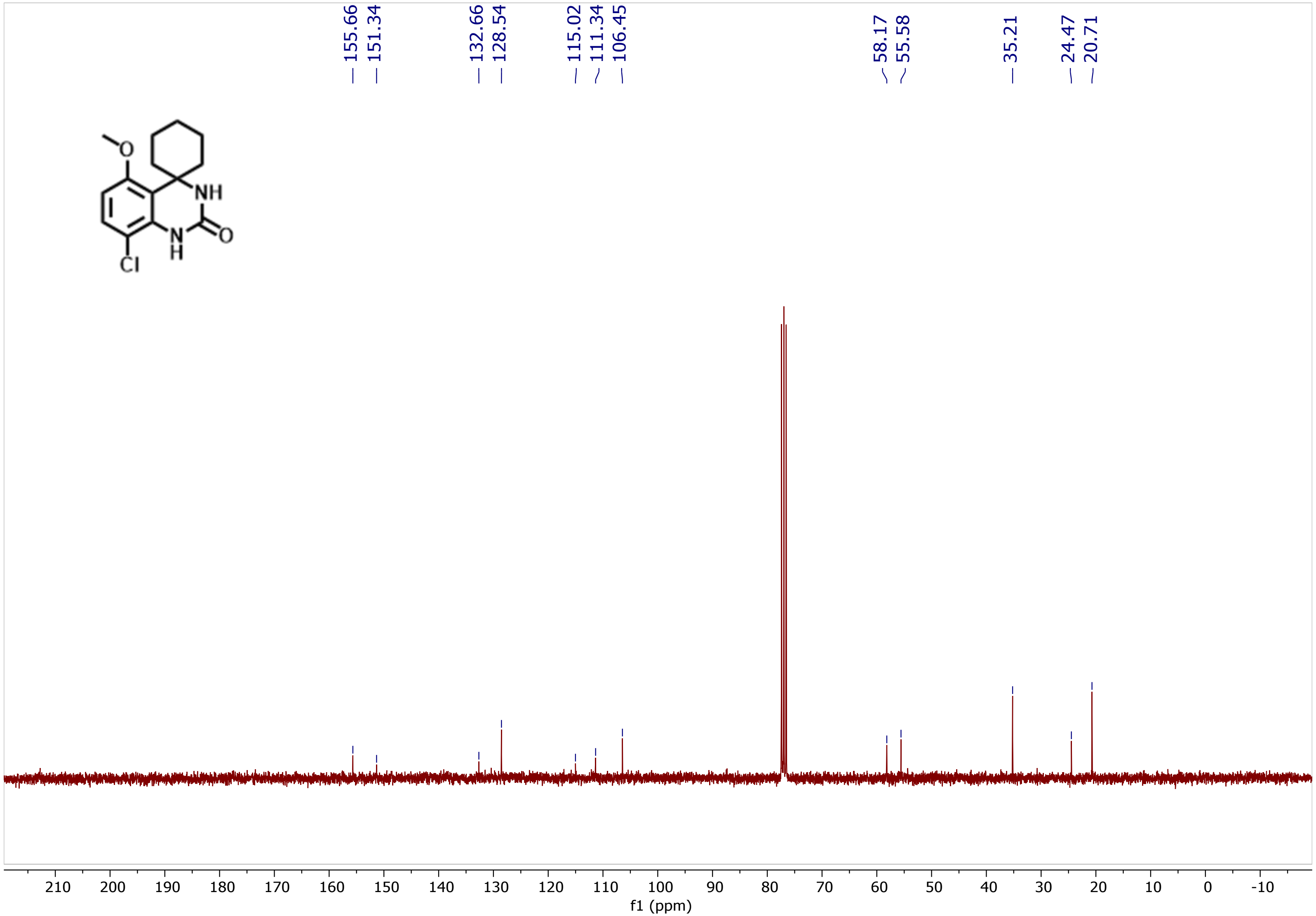

^{1}H spectrum of compound **31**

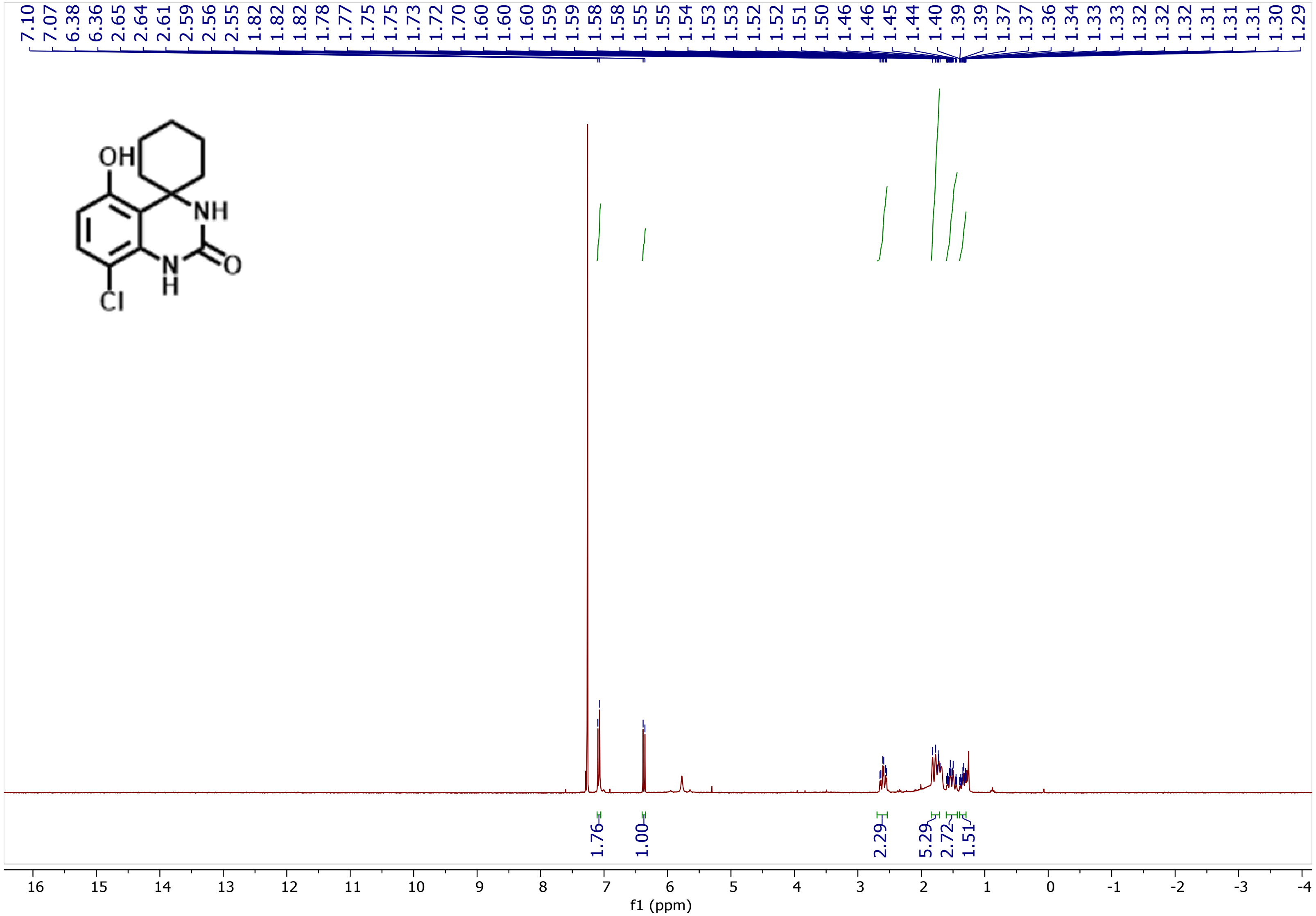

^{13}C spectrum of compound **31**

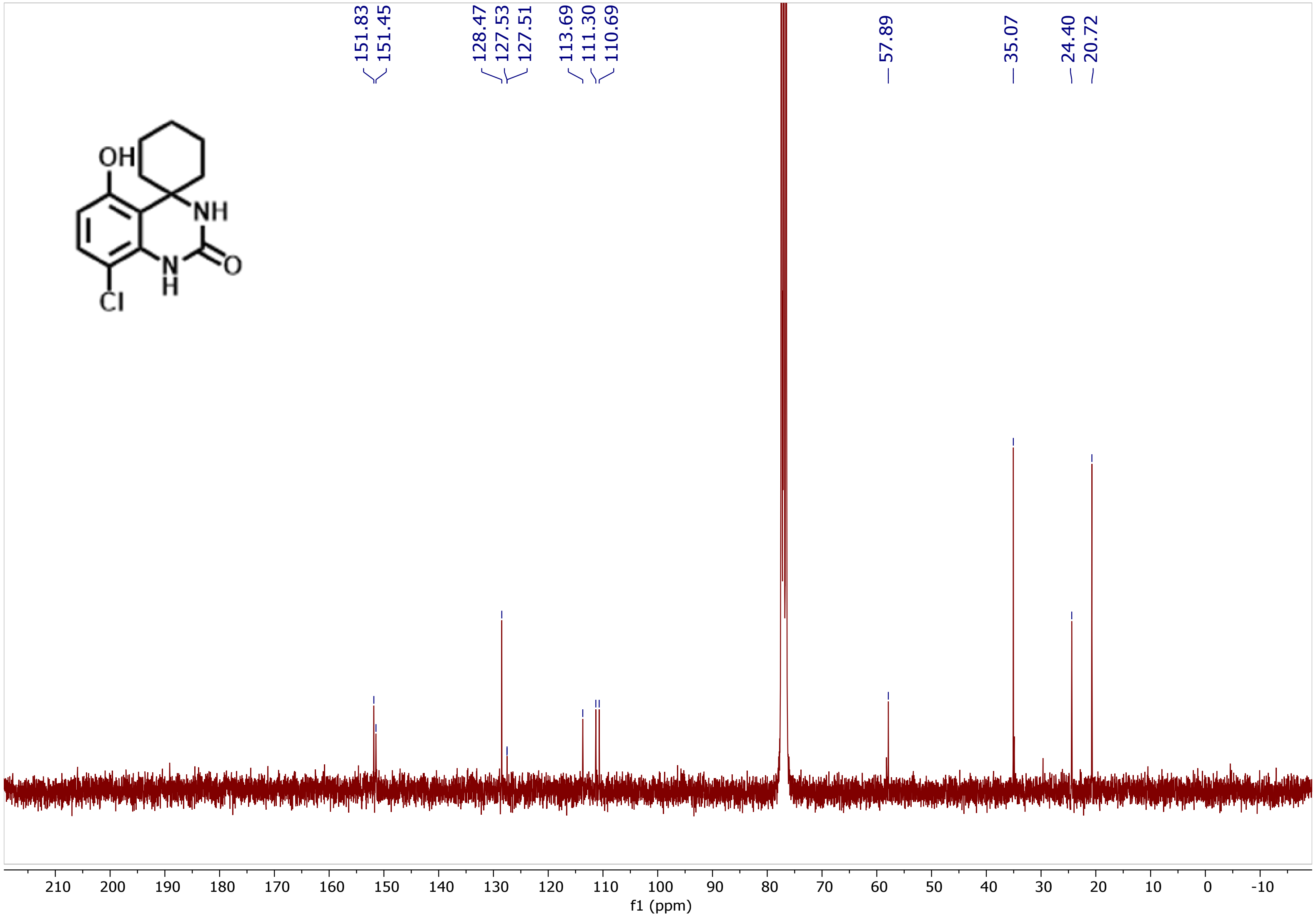

References

1. Bernardelli, P.; Lorthiois, E.; Vergne, F.; Oliveira, C.; Mafroud, A. K.; Proust, E.; Pham, N.; Ducrot, P.; Moreau, F.; Idrissi, M.; Tertre, A.; Bertin, B.; Coupe, M.; Chevalier, E.; Descours, A.; Berlioz-Seux, F.; Berna, P.; Li, M. Spiroquinazolinones as novel, potent, and selective PDE7 inhibitors. Part 2: Optimization of 5,8-disubstituted derivatives. *Bioorg Med Chem Lett* **2004,** 14, 4627-31.
2. Bordoli, L.; Kiefer, F.; Arnold, K.; Benkert, P.; Battey, J.; Schwede, T. Protein structure homology modeling using SWISS-MODEL workspace. *Nature Protocols* **2009,** 4, 1-13.
3. https://swissmodel.expasy.org/
4. Sun, J.-y.; Kumata, K.; Chen, Z.; Zhang, Y.-d.; Chen, J.-h.; Hatori, A.; Fu, H.-l.; Rong, J.; Deng, X.-y.; Yamasaki, T.; Xie, L.; Hu, K.; Fujinaga, M.; Yu, Q.-z.; Shao, T.; Collier, T. L.; Josephson, L.; Shao, Y.-h.; Du, Y.-f.; Wang, L.; Xu, H.; Zhang, M.-r.; Liang, S. H. Synthesis and preliminary evaluation of novel 11C-labeled GluN2B-selective NMDA receptor negative allosteric modulators. *Acta Pharmacologica Sinica* **2021,** 42, 491-498.
5. Taddio, M. F.; Mu, L.; Castro Jaramillo, C. A.; Bollmann, T.; Schmid, D. M.; Muskalla, L. P.; Gruene, T.; Chiotellis, A.; Ametamey, S. M.; Schibli, R.; Krämer, S. D. Synthesis and Structure–Affinity Relationship of Small Molecules for Imaging Human CD80 by Positron Emission Tomography. *Journal of Medicinal Chemistry* **2019,** 62, 8090-8100.
